# Peak systolic blood pressure during preparticipation exercise testing in 12,083 athletes: age, sex, and workload-indexed values and predictors

**DOI:** 10.3389/fphys.2024.1456331

**Published:** 2024-11-22

**Authors:** Petra Pesova, Bogna Jiravska Godula, Otakar Jiravsky, Libor Jelinek, Marketa Sovova, Katarina Moravcova, Jaromir Ozana, Ivan Ranic, Radek Neuwirth, Roman Miklik, Matej Pekar, Libor Sknouril, Vladimir Tuka, Eliska Sovova

**Affiliations:** ^1^ Department of Exercise Medicine and Cardiovascular Rehabilitation, University Hospital Olomouc and Faculty of Medicine, Palacky University, Olomouc, Czechia; ^2^ Sports Cardiology Center, Agel Hospital Trinec-Podlesi, Trinec, Czechia; ^3^ Faculty of Medicine, Masaryk University, Brno, Czechia; ^4^ Faculty of Medicine, University of Ostrava, Ostrava, Czechia; ^5^ Second Department of Medicine - Department of Cardiovascular Medicine, General University Hospital and First Faculty of Medicine, Charles University, Praha, Czechia

**Keywords:** blood pressure, exercise testing, athletes, SBP/WR slope, SBP/WR ratio

## Abstract

**Aim:**

Assessment of blood pressure during exercise is routine in athletes, but normal values remain equivocal. This study examines the response of systolic blood pressure (SBP) to exercise in a large cohort of athletes and establishes normative values by sex and age.

**Methods:**

Competitive athletes free of cardiovascular disease underwent pre-participation exercise testing on a bicycle ergometer. Resting (SBPrest) and peak blood pressure (SBPpeak), heart rate (HRrest and HRpeak), and power output (WR) were recorded. Workload indexed values were calculated.

**Results:**

The cohort included 12,083 athletes (median age 15 years, 26.9% female). Median peak exercise SBP was similar between sexes, but WR-indexed measures including SBP/WR ratio and SBP/(WR/kg) slope were higher in females (0.9 vs. 0.7, *p* < 0.001; 10.94 vs. 9.52, *p* < 0.001). Univariate analyses revealed significant associations between SBPpeak and several predictors, including sex, age, weight, height, SBPrest, DBPrest, HRrest, HRpeak, and WR (all *p* < .001). Multivariate analysis showed that SBPrest (beta = 0.353, 95% CI [0.541, 0.609], *p* < 0.001), height (beta = 0.303, 95% CI [0.360, 0.447], *p* < 0.001), WR (beta = 0.171, 95% CI [0.029, 0.045], *p* < 0.001), and age (beta = 0.093, 95% CI [0.162, 0.241], *p* < 0.001) were the strongest predictors of SBPpeak.

**Conclusion:**

This study provides reference values for the interpretation of SBP responses to exercise in athletes. Multivariate analyses highlight the complex interplay of factors influencing peak SBP, including SBPrest, height, WR, age, DBPrest, sex, endurance sport category, and weight. In future studies, these findings may inform the development of personalised training strategies and risk stratification models in athletic populations.

## Introduction

Competitive athletes and highly active people engage in physical activity at both competitive and recreational levels. To ensure their safety, pre-participation screening is used to assess their fitness level and identify potential health risks (Jiravska et al., 2023; Ljungqvist et al., 2009; Thompson and Levine, 2006). An essential part of this screening is the measurement of resting blood pressure (BP) and, in selected individuals, also BP during exercise testing (Berge et al., 2015; Petek and Baggish, 2020).

Accurate BP measurement is particularly important in athletes, as abnormal BP responses to exercise can have adverse consequences, such as increased risk of cardiovascular disease, reduced athletic performance, and potential harm to their overall health and well being (Hedman et al., 2019; Caselli et al., 2019). Cardiovascular health screening using BP responses to exercise is a key measure in the evaluation of athletes, not only to determine their eligibility to compete but also to predict potential health risks. Exaggerated BP responses to exercise have been associated with an increased risk of developing hypertension, left ventricular hypertrophy and other cardiovascular abnormalities later in life (Nayor et al., 2023; Miyai et al., 2002; Pesova et al., 2023). Therefore, identification of athletes with abnormal BP responses to exercise is critical for early intervention and management.

The demarcation between typical and exaggerated BP responses is an important factor in determining cardiovascular stress and predicting future health challenges. However, it is not clearly defined in the athlete population (Berge et al., 2015). Traditional methods, such as fixed BP levels or incremental increases per metabolic equivalent (MET), do not adequately capture the complex BP fluctuations that athletes exhibit during exercise (Caselli et al., 2016). Different cardiovascular guidelines have different thresholds for defining abnormal BP responses during exercise for the general population. For peak systolic blood pressure (SBP) during exercise, the American Heart Association (AHA) suggests thresholds of 210 mmHg for men and 190 mmHg for women (Gibbons et al., 2002), emphasizing an approximate increase in SBP of 10 mmHg per MET (Fletcher et al., 2013; Hedman et al., 2021). The European Society of Cardiology (ESC) recommends slightly higher values of 220 mmHg for men and 200 mmHg for women (Williams et al., 2018). In contrast, in athletes the American College of Sports Medicine (ACSM) suggests a unisex threshold of 225 mmHg (Hunter et al., 2023). This variability highlights the complexity of interpreting BP responses during exercise.

The primary aim of this research is to provide normative values for sex- and age-specific groups and to contribute to a better understanding of the normal BP response to exercise in athletes. By establishing reference values for the BP response to exercise in different age and sex groups, our findings may help clinicians to identify athletes who may be at risk and require closer monitoring or intervention. We also sought to explore the complex interplay of factors influencing peak SBP through multivariate analysis, with the aim of developing a predictive model for peak SBP in athletes. These findings may inform the development of personalized training strategies and risk stratification models in athletic populations.

## Methods

### Athlete cohort

This retrospective study involved a detailed analysis of pre-participation screening (PPS) data from a comprehensive registry of 13,670 athletes. The data were collected between January 2015 and June 2022 in two sports medicine clinics in the Czech Republic. A total of 1,587 athletes (11.60%) were excluded from the study for the following reasons: 770 (5.60%) underwent non-exercise assessment (ECG and physical examination only), 693 (5.10%) had incomplete exercise data, and 124 (0.90%) had a history of cardiovascular disease or used medications or had other comorbidities that could affect BP response, such as diabetes, and renal disease.

**FIGURE 1:**
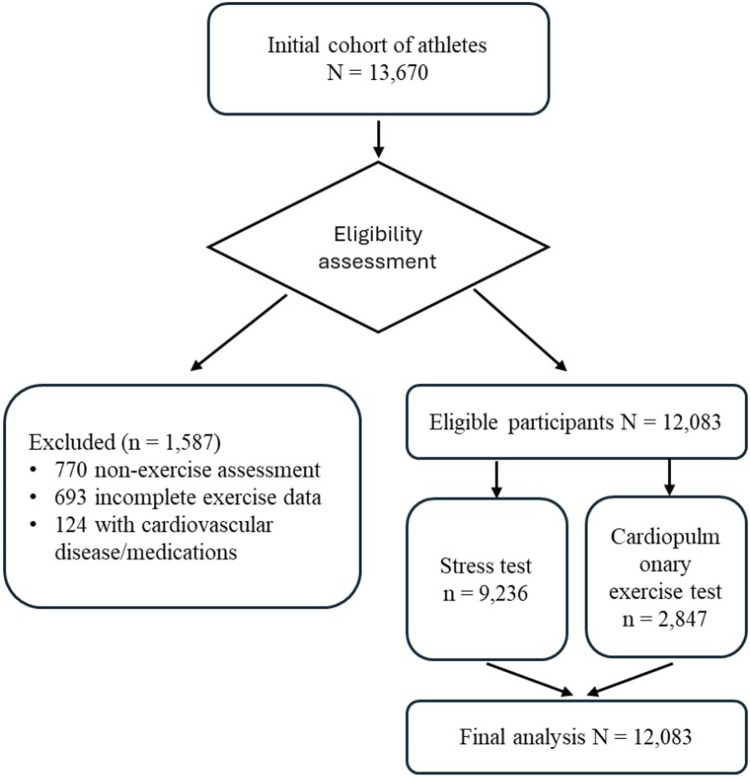
Participant Flow Diagram legend. This flow diagram illustrates the selection process and distribution of participants in the study. From an initial cohort of 13,670 athletes, 1,587 were excluded based on specific criteria. The remaining 12,083 eligible participants underwent either a stress test (n = 9,236) or a cardiopulmonary exercise test (n = 2,847). All 12,083 participants were included in the final analysis. This diagram provides a clear overview of the study population from initial recruitment to final data analysis.

The remaining 12,083 athletes (88.40%) were included in the final analysis, with 9,236 (76.40%) undergoing a stress test to assess exercise tolerance and 2,847 (23.60%) undergoing a cardiopulmonary exercise test (CPET) for a detailed assessment of cardiovascular and pulmonary responses to exercise (Figure 1).

Athlete demographics collected included sex, age, weight, height, BMI, and type of sport, which was categorized as mixed, power, endurance, and skill according to the 2020 European Society of Cardiology guidelines (Pelliccia et al., 2020). This stratified diagnostic approach allowed a multi-level assessment of cardiovascular health and exercise response across a wide range of sporting disciplines.

### Exercise testing

The athletes underwent a progressive maximal exercise test on a cycle ergometer using an individualised ramp protocol. This protocol was chosen to allow for a gradual increase in workload (WR) and to consider the different fitness levels of the athletes. The protocol began with a warm-up period of 3–4 min at WR of 1.0–2.0 W/kg, depending on the athlete’s fitness level. This was followed by a rest period of 1 min or slightly longer to allow the heart rate (HR) to fall below one hundred beats per minute. The actual exercise test then began with a load of 1.5–2.0 W/kg (depending on fitness level and responses observed during warm-up) and the load was increased by 0.25 W/kg every 30 s until exhaustion. The test was stopped by the athletes when they reached maximal effort or if there were other reasons for termination (e.g., ECG evidence of ischaemia or arrhythmia). Cardiopulmonary exercise testing (CPET) was performed in a subcohort of athletes at the request of the athlete, coach, club, or sports federation using the same protocol.

### Parameters measured

A 12-lead electrocardiogram (ECG) was recorded at baseline, during exercise, and immediately after exercise termination. Resting BP was measured after 1 min of rest at the start of the Pre-Participation Screening (PPS), immediately after the baseline ECG was taken. The athlete was seated, and the arm was supported at heart level during the measurements. SBP was measured by auscultation using a calibrated aneroid sphygmomanometer with an appropriately sized cuff based on the athlete’s arm circumference. The cuff was placed on the right arm, with the lower edge approximately 2.5 cm above the antecubital fossa. Peak exercise SBP (SBPpeak) was measured immediately after the athlete stopped cycling at maximum effort. The first audible Korotkoff sound was recorded as the SBP. Diastolic blood pressure (DBP) was not measured at peak exercise because of the unreliability and inaccuracy of DBP measurements during exercise. Only resting (SBPrest) and peak exercise (SBPpeak) values were used for analysis. Heart rate was measured by continuous ECG recording. In addition, perceived exertion using the Borg scale (Williams, 2017; Borg, 1982) and any symptoms experienced by the athletes during the test were recorded.

### Parameters calculated

The following parameters were calculated from the collected data:

1. SBP/WR-slope: This parameter represents the rate of change in systolic blood pressure (SBP) relative to the change in work rate. It is calculated as: SBP/WR-slope = (SBPpeak - SBPrest)/WRpeak The units are mmHg/W, indicating the increase in SBP per unit of work rate. This measure provides insight into the cardiovascular system’s responsiveness to increasing exercise intensity. (Hedman et al., 2021).
2. SBP/(WR/kg) slope: This is the SBP/WR slope indexed to the athlete’s body weight. It is calculated as: SBP/(WR/kg) slope = SBP/WR-slope × body weight (kg) By accounting for body weight, this parameter allows for more standardized comparisons between athletes of different sizes. The units are mmHg/(W/kg). (Bauer et al., 2021).
3. SBPpeak/WR ratio: This ratio reflects the absolute SBP achieved at maximum work rate. It is calculated as: SBPpeak/WR ratio = SBPpeak/WRpeak The units are mmHg/W. This parameter provides a snapshot of the cardiovascular stress at peak exercise relative to the work being performer. (Caselli et al., 2016).

These parameters were chosen for their potential to provide a comprehensive understanding of the SBP response to exercise, considering individual differences in fitness level and body composition. They allow for standardized comparisons across different athletes and align with parameters used in previous studies, facilitating comparison with published data.

### Ethics and institutional review board statement

The study was conducted in accordance with the Declaration of Helsinki, and the protocol was approved by the Ethics Committee of Agel Hospital Trinec Podlesi (approval code: EK 318/22, date: 1.12.2022). Athletes did not provide informed consent to participate in the study due to its retrospective nature; however, data were analysed anonymously. In addition, athletes in both centres gave their consent for their data to be used anonymously for medical research purposes.

### Statistical analysis

Statistical Analysis Due to non-normal data distributions (Shapiro-Wilk test), non-parametric tests were used for all analyses (IBM SPSS Statistics, version 29.0.0.0). Descriptive statistics included means ± SD, medians, and percentiles. The Mann-Whitney U and Kruskal-Wallis tests compared variable distributions between groups, while chi-squared tests assessed proportional differences (significance: *p* < 0.05).

Univariate analyses (Mann-Whitney U, Kruskal-Wallis, Spearman’s rho) examined relationships between predictors and peak systolic blood pressure (SBPpeak). Variables with *p* < 0.1 were included in a multivariate linear regression to explore independent effects on SBPpeak, adjusting for confounders and interactions. The model was built using a hierarchical approach with stepwise selection.

Model performance was evaluated using R-squared, adjusted R-squared, F-test (ANOVA), t-tests, and standardized coefficients (Beta). Predictive accuracy was assessed using mean squared error (MSE) and root mean squared error (RMSE). Assumptions were checked using residual and normal probability plots. Collinearity diagnostics (tolerance, VIF) indicated no severe multicollinearity. Box and scatter plots visualized associations.

## Results

### Cohort characteristics

The study involved 12,083 athletes, all of whom were Caucasian and 26.3% of whom were female. This cohort represents a diverse cross-section of the athletic population, covering a wide range of ages, sports, and performance levels. Athletes were categorised according to the European Society of Cardiology (ESC) 2020 guidelines, which provide a framework for classifying sports based on their dynamic and static components. The majority of participants (74.1%) participated in mixed sports, which combine dynamic and static components, while 16.8% were engaged in endurance sports, 5.8% in power sports, and 3.2% in skill-based sports.

The median age of the athletes was 15 years (interquartile range: 12–19 years) and ranged from 5 to 78 years. This wide age range allows the cardiovascular response to exercise to be analysed across different stages of development and age. The median weight and height were 58.8 kg (interquartile range: 44.0–72.8 kg) and 167 cm (interquartile range: 154–177 cm), respectively. The median BMI was 20.7 kg/m^2^ (interquartile range: 18.0–23.6 kg/m^2^), with a wide range from 10.1 to 41.1 kg/m^2^, reflecting the diversity of body composition among athletes in different sports. Body fat percentage, measured by bioimpedance, was available for a subset of the cohort (28.2%; N = 3,403) and had a median of 14.0% (interquartile range: 11.0%–18.0%), ranging from 3.0% to 42.0%.

The median resting systolic BP was 120 mmHg (interquartile range: 110–130 mmHg), with values ranging from 80 to 185 mmHg. The median resting heart rate was 76 bpm (interquartile range: 69–86 bpm), with a range from 40 to 125 bpm. Table 1 presents detailed demographic data for the entire cohort.

**TABLE 1:** Cohort characteristics.

	N	%	Median (Q1 – Q3)	Min – Max
Total Cohort	12,083			
Center 1	3,414	28.3%		
Center 2	8,669	71.7%		
Sex				
Female	3,175	26.3%		
Male	8,897	73.7%		
ESC 2020 Sport Category				
Mixed	6,259	74.1%		
Endurance	1,423	16.8%		
Power	493	5.8%		
Skill	274	3.2%		
Examination Age (years)	12,083		15 (12–19)	5–78
Weight (kg)	12,083		58.8 (44.0–72.8)	21.1–154.6
Height (cm)	12,083		167 (154–177)	110–211
BMI (kg/m^2^)	12,083		20.7 (18.0–23.6)	10.1–41.1
Bioimpedance Body Fat (%)	3,403		14.0 (11.0–18.0)	3.0–42.0
SBPrest (mmHg)	12,083		120 (110–130)	80–185
DBPrest (mmHg)	12,083		70 (70–80)	40–120
HRrest (bpm)	12,083		76 (69–86)	40–125

Note: The table presents continuous variables as median values with interquartile ranges due to non-normal distributions, alongside the full range expressed as minimum and maximum values. Percentages reflect the proportion of athletes within each category, based on those who underwent stress tests and CPET (N = 12,083), with categorical counts indicated. Age is reported in years; weight in kilograms (kg); height in centimeters (cm); BMI, in kg/m^2^; bioimpedance body fat as a percentage (%); resting blood pressure in millimeters of mercury (mmHg); and resting heart rate in beats per minute (bpm). For a detailed breakdown of age categories and the number of participants in each, please refer to Table 2.

BMI, body mass index; CPET, cardiopulmonary exercise test; DBPrest -resting diastolic blood pressure, ESC, European Society of Cardiology; HRrest–resting heart rate; SBPrest–resting systolic blood pressure.

### Blood pressure response

Table 2 summarises the cardiovascular response to exercise by sex, sport, and age categories. Although females and males had the same median SBPpeak (160 mmHg), females exhibited a narrower interquartile range (Q1-Q3: 145–170) compared to males (Q1-Q3: 145–180), with a statistically significant difference (*p* < .001).

**TABLE 2:** Cardiovascular response to exercise: a comparative analysis of blood pressure, heart rate, and power output among athletes across sex, sport, and age categories.

		SBPpeak		HRpeak		WRpeak per Kilogram	
		Median (Q1-Q3)	*P*-value	Median (Q1-Q3)	*P*-value	Median (Q1-Q3)	*P*-value
Total (N = 12,083)		160 (145–180)		186 (178–194)		4.0 (3.5–4.5)	
Sex	Female	160 (145–170)	<.001	183 (173–191)	<.001	3.5 (3.0–3.8)	<.001
	Male	160 (145–180)		188 (179–195)		4.2 (3.7–4.7)	
ESC2020 Sport Category	Mixed	150 (140–165)	<.001	189 (182–196)	0.799	4.2 (3.5–4.7)	<.001
	Skill	150 (140–160)		189 (182–195)		4.0 (3.5–4.5)	
	Endurance	150 (140–160)		189 (181–196)		4.0 (3.5–4.5)	
	Power	150 (140–165)		189 (182–196)		4.0 (3.5–4.5)	
Age Category	≤9 (n = 588)	135 (130–145)	<.001	186 (180–194)	<.001	3.7 (3.2–4.2)	<.001
	10–11 (n = 1893)	140 (135–150)		190 (182–197)		4.0 (3.5–4.3)	
	12–14 (n = 3,340)	150 (140–165)		190 (183–197)		4.1 (3.5–4.7)	
	15–17 (n = 2,219)	170 (160–180)		189 (181–194)		4.5 (3.8–5.0)	
	18–30 (n = 2,315)	175 (160–190)		184 (178–191)		4.0 (3.5–4.6)	
	31–40 (n = 413)	185 (170–195)		177 (169–184)		3.8 (3.2–4.3)	
	41–50 (495)	185 (170–200)		170 (160–180)		3.3 (2.5–4.0)	
	51–60 (n = 548)	185 (165–200)		159 (146–171)		2.5 (1.8–3.2)	
	61–70 (n = 219)	180 (170–200)		145 (129–161)		2.0 (1.6–2.9)	
	>70 (n = 53)	170 (150–190)		146 (128–173)		1.7 (1.3–2.4)	

Note: This table summarizes the medians and interquartile ranges (Q1-Q3) for peak systolic blood pressure (SBPpeak), heart rate (HRpeak), and WRpeak per kilogram across different athlete demographics and sport classifications. The *p*-values were calculated using the Mann-Whitney U test to assess the statistical significance of differences between groups, with a threshold of *p* < 0.05 indicating significance. Abbreviations: ‘ESC, 2020′refers to the ESC, guidelines used to categorize sports; SBP, is measured in millimeters of mercury (mmHg), HR, in beats per minute (bpm), and Peak Work Rate per Kilogram in watts per kilogram (W/kg).

Across ESC 2020 sport categories, median SBPpeak was similar at 150 mmHg, with slightly different interquartile ranges. The highest Q3 level (165 mmHg) was noted in power and mixed sports. Despite comparable medians, SBPpeak differed significantly between the sport categories (*p* <.001).

Median SBPpeak increased with age, from 135 mmHg (Q1-Q3: 130–145) in the ≤9 years group to 185 mmHg (Q1-Q3: 165–200) in the 51–60 years group, before decreasing to 170 mmHg (Q1-Q3: 150–190) in the >70 years group.

HRpeak remained relatively stable across age groups, with medians ranging from 186 bpm (Q1-Q3: 180–194) in the youngest group to 190 bpm (Q1-Q3: 182–197) in the 10–14 years group, followed by a gradual decline to 146 bpm (Q1-Q3: 128–173) in the >70 years group.

Peak work rate progressively decreased with increasing age, from a median of 4.0 W/kg (Q1-Q3: 3.5–4.5) in the total population to 1.7 W/kg (Q1-Q3: 1.3–2.4) in the >70 years group.

Statistical analysis showed significant differences (*p* < .001) for all comparisons, except for HRpeak between sport categories (*p* = 0.799), indicating that age, sex, and sport type significantly influenced the cardiovascular response to exercise, with peak HRpeak being the only variable that does not differ between sport categories.

### Peak systolic blood pressure correlation

Univariate analyses were conducted to examine the relationship between each predictor variable and SBPpeak (Supplementary Table S1 in the Supplementary Materials.). The analyses revealed significant associations between SBPpeak and several predictors, including sex, age, weight, height, SBPrest, DBPrest, HRrest, HRpeak, and WR (all *p* < .001). The type of sport was also significantly associated with SBPpeak (*p* < 001), with athletes engaged in mixed and endurance sports showing significant differences in SBPpeak compared to other categories (*p* < 001).

Our study used multivariate regression analysis to further examine the relationship between SBPpeak and the significant predictors identified in the univariate analyses. The results showed that WR, SBPrest, height, and age were the strongest predictors of SBPpeak (results in Table 3).

**TABLE 3:** Multivariate regression analysis of Peak Systolic Blood Pressure.

Predictor	Coefficient (B)	Standard Error	Standardized Coefficients (Beta)	t-value	Statistical significancy (*p*-value)	95% Confidence Interval for B		Collinearity Statistics	
						Lower Bound	Upper Bound	Tolerance	VIF
(Constant)	18,573	4,217		4,404	<.001	10,306,326	26,84		
SBPrest	0,575	0,017	0,353	33,147	<.001	0,541	0,609	0,529	1,889
Height	0,403	0,022	0,303	18,258	<.001	0,360	0,447	0,218	4,579
Work Rate	0,037	0,004	0,171	9,122	<.001	0,029	0,045	0,171	5,862
Age	0,202	0,020	0,093	10,054	<.001	0,162	0,241	0,697	1,434
DBPrest	0,055	0,019	0,027	2,900	0,004	0,018	0,092	0,679	1,474
Sex	−0,910	0,368	−0,022	−2,471	0,014	−1,632	−0,188	0,761	1,314
Endurance	−1,475	0,588	−0,030	−2,508	0,012	−2,628	−0,322	0,421	2,378
Weight	−0,086	0,026	−0,069	−3,325	0,001	−0,136	−0,035	0,141	7,091
Mixed	−0,861	0,502	−0,020	−1,713	0,087	−1,845	0,124	0,420	2,379
HRpeak	−0,022	0,014	−0,014	−1,555	0,120	−0,049	0,006	0,754	1,326
HRrest	−0,012	0,012	−0,008	−0,999	0,318	−0,036	0,012	0,916	1,092

Note: The model includes several predictors: WRpeak, SBPrest, examination age, height, weight, HRrest, sex (1 for male, 0 for female), DBPrest, endurance sport category (1 for endurance, 0 for other categories), and HRpeak. The standardised coefficients (Beta) and their significance (*p*-values) are presented for each predictor, with the dependent variable being SBPpeak.

The standardised coefficients (Beta) from the analysis indicate the relative importance of each variable in predicting peak SBP. SBPrest had the highest standardised coefficient (Beta = 0.353, 95% CI [0.541, 0.609], *p* < .001), followed by height (Beta = 0.303, 95% CI [0.360, 0.447], *p* < .001), WR (Beta = 0.171, 95% CI [0.029, 0.045], *p* < .001), and age (Beta = 0.093, 95% CI [0.162, 0.241], *p* < .001). These results suggest that higher resting systolic blood pressure, taller stature, higher maximal power output, and increasing age are associated with higher SBPpeak during exercise.

**FIGURE 2:**
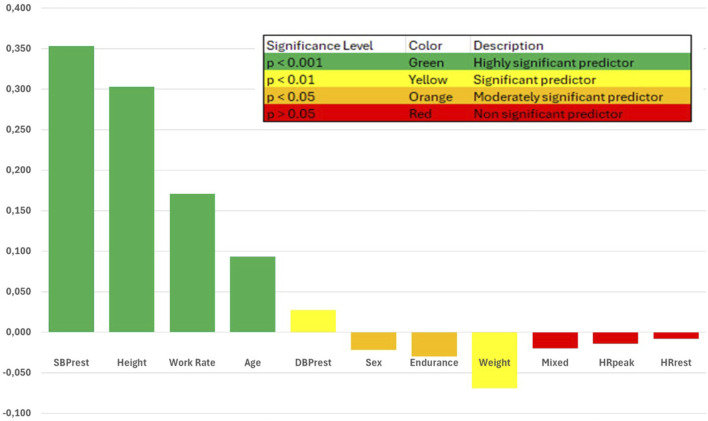
Standardized Coefficients of Predictors of Peak Systolic Blood Pressure in Athletes. legend This figure displays the standardized coefficients (β) from a multivariate regression analysis examining the relationship between peak systolic blood pressure (SBPpeak) and several demographic and resting parameters in a cohort of athletes undergoing preparticipation testing. The standardized coefficients represent the relative strength and direction of each predictor’s association with SBPpeak, with larger absolute values indicating a stronger relationship. Green bars indicate highly significant positive predictors (*p* < .001), yellow bars indicate significant positive predictors (*p* < 0.01 and *p* < 0.05), and red bars indicate non-significant predictors (*p* > 0.05). Resting systolic blood pressure, height, workrate, and age were the strongest predictors of SBPpeak in this athlete cohort.

Other factors, such as weight (Beta = −0.069, 95% CI [-0.136, −0.035], *p* = 0.001), endurance sport category (Beta = −0.030, 95% CI [-2.628, −0.322], *p* = 0.012), male sex (Beta = −0.022, 95% CI [-1.632, −0.188], *p* = 0.014), and DBPrest (Beta = 0.027, 95% CI [0.018, 0.092], *p* = 0.004), also showed significant, although weaker, associations with SBPpeak. For clarity, the standardised coefficients (Beta) are shown in Figure 2.

R^2^, the coefficient of determination, which represents the proportion of variance in the dependent variable that can be explained by the independent variables. Here, R^2^, and also adjusted R^2^ is equall to0.495, meaning that 49.5% of the variance in SBPpeak can be explained by the predictors included in the model.

Analysis of Variance (ANOVA) indicates that the regression model, is statistically significant (F(11, 8,396) = 748.840, *p* < 0.001). This means that the model explains a significant amount of the variance in SBPpeak, and there is a linear relationship between the predictors and the dependent variable.

Based on the multivariate analysis, we propose the following formula for predicting SBPpeak:

$$\begin{array}{ll} \mathrm{Peak}\;\mathrm{SBPpredicted} & =18.573+\left(0.575\times \mathrm{SBPrest}\right)+\left(0.403\times \mathrm{Height}\right)\\ & +\left(0.037\times \mathrm{Work}\;\mathrm{Rate}\right)+\left(0.202\times \mathrm{Age}\right)\\ & +\left(0.055\times \mathrm{DBPrest}\right)-\left(0.910\;\mathrm{for}\;\mathrm{Male}\;\mathrm{Sex}\right)\\ & -\left(1.475\;\mathrm{for}\;\mathrm{Endurance}\;\mathrm{Sport}\;\mathrm{Category}\right)-\\ & \left(0.086\times \mathrm{Weight}\right) \end{array}$$

To assess the performance of the prediction model, we calculated the mean squared error (MSE) and the root mean squared error (RMSE). The MSE was found to be 201.8, and the RMSE, which represents the average prediction error in the same units as peak SBP (mmHg), was 11.57 (SD 8.24). An RMSE of 11.57 mmHg indicates that, on average, the predicted peak SBP values differ by approximately 12 mmHg from the observed values, providing a measure of the goodness of fit of the model.

### Indexed blood pressure response

Table 4 presents our indexed results, providing a comprehensive breakdown of the SBP response when normalised for WR and body weight. Female athletes had a median SBPpeak/WR ratio of 0.9 (Q1-Q3: 0.7–1.1) compared to 0.7 (Q1-Q3: 0.6–0.9) for males, suggesting a more pronounced cardiovascular response to exercise intensity in females (*p* < .001). The SBP/WR slope followed a similar pattern, with a median of 0.21 (Q1-Q3: 0.15–0.27) for females and 0.17 (Q1-Q3: 0.13–0.23) for males, reinforcing the notion of sex-based physiological differences (*p* < .001). Looking at the SBP/(W/kg) slope, a value that integrates both power output and the athlete’s weight, we observed a median of 10.94 (Q1-Q3: 8.11–15.15) for females and 9.52 (Q1-Q3: 6.73–13.33) for males, which was statistically significant (*p* < .001).

**TABLE 4:** Overview of calculated exercise parameters.

	Total Cohort	Female	Male	*P*-value
SBPpeak/WR Ratio	0.8 (0.6–0.9)	0.9 (0.7–1.1)	0.7 (0.6–0.9)	<.001
SBP/WR Slope	0.18 (0.13–0.24)	0.21 (0.15–0.27)	0.17 (0.13–0.23)	<.001
SBP/(W/kg) Slope	10.00 (7.14–13.73)	10.94 (8.11–15.15)	9.52 (6.73–13.33)	<.001

Note: This table presents the median values with the 25th and 75th percentiles in parentheses for exercise-related parameters among the total cohort and by sex. The Mann-Whitney U test was employed to determine the statistical significance of differences between female and male athletes, denoted by the *P*-values. A *P*-value less than .001 indicates a highly significant difference. Abbreviations: SBPpeak, peak systolic blood pressure; WR, work rate; W/kg, watts per kilogram of body weight.

The key findings from Table 4 highlight the sex differences in indexed SBP response to exercise. Females consistently had higher median values for SBPpeak/WR ratio, SBP/WR slope, and SBP/(W/kg) slope compared to males, indicating a more pronounced cardiovascular response to increasing WR relative to body weight. These results suggest that females may experience a greater increase in SBP for a given increase in WR, even when differences in body weight are considered.

**FIGURE 3:**
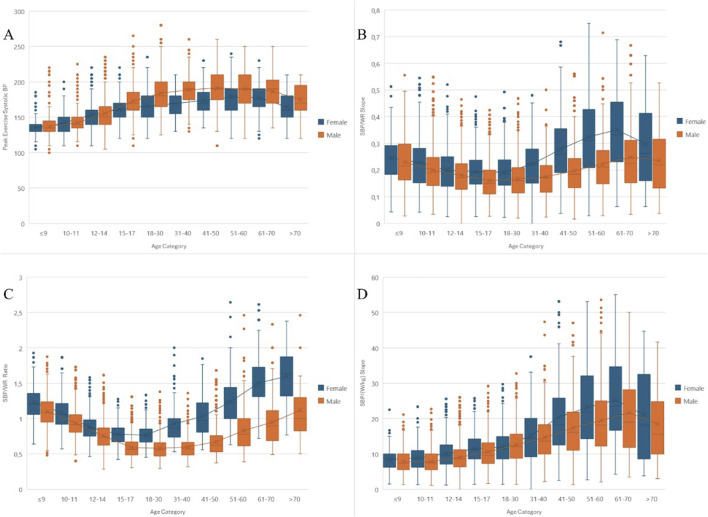
Comprehensive Analysis of Systolic Blood Pressure Responses Across Age and Sex in Athletes During Exercise. General Legend for All Panels: Each panel displays clustered box plots of raw or indexed systolic blood pressure (SBP) responses, categorized by age and sex. Within each box plot, the “X” marks the mean, while the median is represented by a line. Outliers are shown as individual dots. Linear trends connecting the means for both sexes across age categories illustrate changes in SBP response patterns. Specific Legends for Each Panel: Panel **(A)** Shows the peak exercise systolic blood pressure (SBPpeak) across different age groups, separated by sex, highlighting how cardiovascular responses vary during exercise. Panel **(B)** Illustrates the SBP/WR slope, indicating the rate of change in SBP relative to workload increases. It shows a steeper increase in SBP with increasing workload, particularly in females across all age groups. Panel **(C)** Focuses on the SBP/WR ratio, which quantifies the normalized systolic blood pressure response to workload. This panel points to higher values in females across all age groups, suggesting a more pronounced response. Panel **(D)** Presents the SBP/(W/kg) slope, showing SBP response per unit change in workload indexed to body weight. Differences are noted between sexes and across age categories, with a trend of increasing slope in older age groups.

The differences observed in the cardiovascular response to exercise are visually depicted in Figure 3 – panel A–D. Figure 3Afig3 illustrates the variation in SBPpeak achieved by sex and by age group, showing a general increase with age up to the 51–60 age group, followed by a slight decrease in the older age groups. Figure 3B shows the SBP/WR slope, which represents the rate of change in SBP relative to the change in WR. Females have higher median SBP/WR slopes across all age groups, with a notable increase in slope values in the older age categories for both sexes. Figure 3C focuses on the SBP/WR ratio by sex and age, showing consistently higher median values in women compared to men across all age groups. The SBP/WR ratio also tends to increase with age, particularly in the older age groups. Figure 3D displays the SBP/(W/kg) slope, which assesses the systolic blood pressure response per unit change in workload indexed to body weight. This panel highlights significant variations in cardiovascular efficiency in response to exercise stress, with females exhibiting higher median SBP/(W/kg) slopes compared to males across various age categories. Notably, the data also illustrate an increasing trend in SBP/(W/kg) slope values with advancing age, suggesting that older age groups may experience a greater increase in SBP relative to workload per kilogram of body weight, emphasizing the impact of age on cardiovascular response during exercise.

Detailed information on SBPpeak and indexed parameters for different sexes and age groups can be found in Supplementary Tables S2, S3 in the Supplementary Materials.

## Discussion

Our study provides new insights into the complex interplay of physiological factors influencing the BP response to exercise, particularly when indexed to WR and body weight. Sex differences in the indexed SBP response are evident, with females showing a more pronounced cardiovascular response to exercise intensity compared with males. The age-related trends in indexed SBP response suggest an increasing cardiovascular demand with advancing age, particularly in the older age groups. These findings highlight the importance of considering not only the absolute values of BP response but also how these values are indexed to exercise intensity and how they vary between sex and age groups.

Our findings are consistent with previous studies reporting age- and sex-specific reference values for the indexed SBP response during bicycle ergometry in the general population (Hedman et al., 2021; Hedman et al., 2020). Compared to the Hedman cohort, our cohort had a significantly higher level of fitness (4.2 W/kg compared to 2.8 W/kg for men and 3.5 W/kg compared to 2.08 W/kg for women) and a higher achieved mean HRpeak. Mean BPrest and HRrest were comparable. All age subgroups of our athletes had lower WR indexed SBP values (SPB/WR ratio and SPB/WR slope) than those published by Hedman, which may be due to the higher WRpeak achieved. This contrasts with the higher SPB/WR slope achieved by the female athletes studied by Bauer (Bauer et al., 2021; Bauer et al., 2020).

The sex differences in indexed SBP response to exercise may be attributed to several factors. Firstly, females may have greater peripheral vascular resistance during exercise compared to males (Hedman et al., 2021), which is supported by experimental studies suggesting differences in the balance between HR, stroke volume, and vascular resistance between sexes, as well as differences in the exercise pressor reflex. These differences may be related to hormonal factors, such as oestrogen levels, which have been shown to influence vascular function and BP regulation (Joyner et al., 2016). Additionally, sex differences in autonomic control of the cardiovascular system during exercise, with females exhibiting greater parasympathetic withdrawal and sympathetic activation compared to males, may contribute to the observed differences in BP response (Samora et al., 2019).

Another explanation for the observed sex differences in indexed SBP response to exercise may be related to differences in body composition between males and females. Females typically have a higher percentage of body fat compared to males, which means that for the same body weight, they have lower muscle mass. In a subset of our cohort (28.2%; N = 3,403) for which body fat percentage data was available, we found that females had a significantly higher median body fat percentage of 22.5% (interquartile range: 18.0%–27.0%) compared to males, who had a median of 13.0% (interquartile range: 11.0%–16.9%) (*p* < .001). If we could index SBP response by muscle mass instead of total body weight, the WR/kg would likely be higher for females than males, potentially leading to more comparable SBP values for a given WR/kg of muscle mass. However, due to the limited availability of body composition data in our cohort, we were unable to fully explore this hypothesis. Future studies with comprehensive body composition assessments are needed to better understand the impact of muscle mass on sex differences in SBP response to exercise.

Our study also highlights the importance of considering age when interpreting BP responses to exercise in athletes. The age-related trends in the indexed SBP response suggest an increasing cardiovascular demand with age, particularly in the older age groups. Elderly athletes are not immune to the effects of arterial aging. Lower arterial compliance leads to higher pulse wave velocity, which increases the summation of antegrade and retrograde pulse waves, increasing BP in central arteries. This finding is consistent with previous studies reporting a decline in exercise tolerance with age (Petek and Baggish, 2020). These age-related changes in BP response to exercise underscore the need for age-specific reference values in athletic populations.

In our study, multivariate regression analysis identified several factors contributing to SBPpeak during exercise, with SBPrest, height, WR, and age being the strongest predictors. These findings are consistent with previous studies that have highlighted the influence of resting blood pressure, anthropometric measures, exercise capacity, and age on the cardiovascular response to exercise (Hedman et al., 2019; Caselli et al., 2016; Hedman et al., 2021). However, our study extends these findings by providing a comprehensive predictive model that includes additional factors such as weight, endurance sport category, sex, and DBPrest. The inclusion of these variables in the model underscores the complex interplay of factors influencing the cardiovascular response to exercise in athletes.

The proposed formula for predicting SBPpeak based on these factors may serve as a valuable tool for estimating peak systolic blood pressure during exercise in athletes. While previous studies have proposed threshold values for SBPpeak based on sex and age (Caselli et al., 2016; Williams et al., 2018), our predictive model offers a more individualized approach by considering a broader range of relevant variables. This approach may help clinicians and coaches to identify athletes at risk of exaggerated blood pressure responses and to develop personalized training and management strategies. However, it is important to note that further validation of this predictive model in independent cohorts of athletes is necessary to assess its generalizability and clinical utility.

Our findings align with recent research highlighting the importance of comprehensive cardiovascular evaluations in athletes. Palermi et al. (2022) demonstrated that questionnaire-based pre-participation screening algorithms (PPSAs) may miss up to 24% of cardiovascular abnormalities, including 28% of high blood pressure cases and 19% of ECG abnormalities (Palermi et al., 2022). This underscores the value of our more detailed approach, which includes exercise stress testing, in assessing cardiovascular health in athletes.

Caselli et al. (2016) proposed a threshold value for SBPpeak of 220 mm Hg for male athletes and 200 mm Hg for female athletes, corresponding to the 95th percentile. Although these thresholds are comparable to our cohort, it is important to note that the Caselli study mainly included athletes with a mean age of 25 ± 6 years. In our study, the subgroups of men aged 30–60 years and women aged 40–70 years exceeded these thresholds. This discrepancy highlights the need for age-specific reference values in athletic populations.

In another study, Caselli et al. reported a higher risk of future arterial hypertension in athletes with an exaggerated BP response (Caselli et al., 2019). It seems that highly fit athletes are able to achieve physiologically higher BP levels during exercise testing. Surprisingly, the male athletes in our cohort did not achieve a higher SBPpeak compared to the sedentary subjects in the Hedman cohort, but the female athletes did. Compared with the Caselli cohort of Olympic athletes, we reported lower SBPpeak values in both sexes. These differences may be due to the different fitness levels and age distributions of the cohorts studied.

Our results are consistent with those of Biffi et al. (2022), who found that about 7% of a corporate population showed significantly reduced cardiorespiratory fitness (CRF) when assessed by exercise stress testing. They also reported that individuals with lower levels of CRF had higher resting and/or peak exercising BP values after adjusting for co-variables (Biffi et al., 2022). This supports our findings on the complex interplay between fitness levels and blood pressure responses to exercise.

A common question is whether the general population and athletes require different exercise test thresholds. A SBP of 250 mmHg is usually considered the threshold for exercise testing termination for safety reasons (Schultz et al., 2017; Albouaini et al., 2007; Löllgen and Leyk, 2018). However, young athletes, particularly males, may exceed this threshold. Therefore, the decision to stop exercise testing should be made on an individual basis, taking into account the athlete’s age, sex, fitness level, and cardiovascular risk profile (Fletcher et al., 2013; Mazaheri et al., 2021).

Recent advancements in ECG interpretation techniques, as reviewed by Smaranda et al. (2024), suggest promising avenues for future research (Smaranda et al., 2024). The application of artificial intelligence (AI), particularly machine learning and deep learning algorithms, in ECG analysis could potentially enhance our ability to detect complex cardiac patterns and differentiate between physiological adaptations and pathological changes in athletes’ hearts. This could be especially valuable in improving the accuracy and efficiency of pre-participation examinations.

Our study has several limitations that should be acknowledged. Firstly, our exclusion criteria, while necessary to ensure data quality and focus on healthy athletes, may introduce some bias. We excluded 11.60% of the initial cohort due to non-exercise assessment, incomplete exercise data, or history of cardiovascular disease and medication use. This exclusion, particularly of those with cardiovascular issues or incomplete data, could potentially lead to an underestimation of BP responses in the athletic population as a whole. However, the relatively low percentage of exclusions suggests that our sample remains largely representative.

Secondly, there was an over-representation of younger athletes and male athletes and a lower representation of endurance and power athletes in our cohort. This may limit the generalisability of our findings to the wider athletic population. Thirdly, information on risk factors such as smoking and the use of performance-enhancing substances was not available, which may have influenced the observed BP responses. Fourthly, we used self-reported data on existing cardiovascular disease or medication, which may be subject to recall bias and fear of obtaining sport clearance.

We acknowledge that information on smoking, supplement use, and dietary patterns would indeed provide valuable context for cardiovascular health. The lack of this data in our retrospective registry is a limitation of our study, and we suggest these factors as important areas for future research.

Another limitation of our study is that we did not directly measure the hydration status of the athletes. Hydration status can potentially influence blood pressure responses during exercise, and this factor may have contributed to some of the variability observed in our results. Previous studies have shown that dehydration can lead to increased cardiovascular strain during exercise, potentially resulting in higher blood pressure responses (Cheuvront et al., 2010). Conversely, hyperhydration has been associated with reduced cardiovascular strain and potentially lower blood pressure responses during exercise (Mora-Rodríguez et al., 2015).

The impact of hydration status on blood pressure behavior during exercise is complex and can vary based on factors such as the degree of dehydration, environmental conditions, and individual physiological characteristics (Kenefick and Cheuvront, 2012). For instance, mild dehydration (1%–2% body mass loss) has been shown to increase heart rate and reduce stroke volume during exercise, which could indirectly affect blood pressure responses (González-Alonso et al., 1985).

However, recent research by Giddings et al. (2024) suggests that the impact of mild dehydration on central blood pressure and pulse wave velocity may be less significant than previously thought, at least in young, healthy individuals (Giddings et al., 2024). Their study found no significant relationship between changes in hydration status and pulse wave velocity or central diastolic blood pressure, although a significant relationship was observed with central systolic blood pressure. These findings indicate that the effects of hydration on blood pressure responses may be nuanced and potentially age-dependent.

Despite this limitation, our large sample size and the randomness of hydration states across participants likely mitigated any systematic bias due to hydration status. However, the potential influence of hydration on individual blood pressure responses should be considered when interpreting our results, particularly in cases of extreme values or outliers.

Future studies should aim to address these limitations by using standardised exercise protocols, collecting more comprehensive data on cardiovascular risk factors, ensuring a more balanced representation of athlete subgroups, and validating our findings in independent cohorts. In addition, longitudinal studies are warranted to investigate the prognostic value of indexed BP responses in predicting future cardiovascular outcomes in athletic populations.

## Conclusions

In conclusion, this study provides normative data of SBPpeak for sex- and age-specific groups and contributes to a better understanding of the normal BP response to exercise in athletes. The reference values and prediction model developed in this study may serve as useful tools to identify athletes subjected to higher cardiovascular stress during exercise and to guide training strategies to mitigate the consequences of high BP during exercise. These findings have potential clinical implications for the pre-participation screening and management of athletes. By identifying athletes with exaggerated BP responses, clinicians may be able to detect masked hypertension and to provide targeted interventions, such as lifestyle modifications or pharmacological treatment, to reduce the risk of future cardiovascular events. In addition, the age- and sex-specific reference values may help to develop individualised training plans that optimise performance while minimising cardiovascular stress.

However, further research is needed to fully understand the BP responses during different types of physical activity and to validate our findings in independent cohorts. Future studies should investigate the long-term prognostic value of indexed BP responses in predicting cardiovascular outcomes in athletic populations and explore the mechanisms underlying the observed sex differences in BP responses.

In summary, this study provides a basis for the use of age- and sex-specific reference values and prediction models in the cardiovascular assessment of athletes. The potential clinical implications and future research directions highlighted here underscore the importance of continued efforts to optimise cardiovascular health and performance in athletic populations.

## Data Availability

The raw data supporting the conclusions of this article will be made available by the authors, without undue reservation.
