# Supplementary material for: Peak systolic blood pressure during preparticipation exercise testing in 12,083 athletes: age, sex, and workload-indexed values and predictors
Source: Front Physiol. 2024 Nov 22;15:1456331. doi: 10.3389/fphys.2024.1456331 (PMC11621218; doi:10.3389/fphys.2024.1456331)
Supplement: Supplementary file 1 [file Table1.docx]

**Table S1.** Univariate Analyses of Predictors of Peak Systolic Blood Pressure (SBPpeak) in Athletes

| Predictor Variable | Variable Type | Statistical Test | Test Statistic | P-value |
| --- | --- | --- | --- | --- |
| Sex | Categorical | Mann-Whitney U | 12889643 | < .001 |
| Sports category | Categorical | Kruskal-Wallis | 30.208 | < .001 |
| Mixed | Categorical | Mann-Whitney U | 6337837 | < .001 |
| Skill | Categorical | Mann-Whitney U | 1099913 | 0.765 |
| Endurance | Categorical | Mann-Whitney U | 4506010 | < .001 |
| Power | Categorical | Mann-Whitney U | 1948705 | 0.987 |
| Age | Continuous | Spearman's rho | 0.648 | < .001 |
| Weight | Continuous | Spearman's rho | 0.729 | < .001 |
| Height | Continuous | Spearman's rho | 0.702 | < .001 |
| SBPrest | Continuous | Spearman's rho | 0.691 | < .001 |
| DBPrest | Continuous | Spearman's rho | 0.502 | < .001 |
| HRrest | Continuous | Spearman's rho | -0,047 | < .001 |
| HRpeak | Continuous | Spearman's rho | -0,251 | < .001 |
| WR | Continuous | Spearman's rho | 0.609 | < .001 |

Note:
This table presents the results of univariate analyses examining the relationship between various predictor variables and peak systolic blood pressure (SBPpeak) in a cohort of athletes. Non-parametric tests were used due to the non-normal distribution of the data. Categorical variables were assessed using the Mann-Whitney U test (for binary variables) or the Kruskal-Wallis test (for variables with more than two categories). Continuous variables were evaluated using Spearman's rank correlation coefficient (rho). The "Sports category" variable was further analyzed by comparing each category (Mixed, Skill, Endurance, and Power) to all other categories combined using the Mann-Whitney U test. P-values < 0.05 were considered statistically significant.
The univariate analyses revealed significant associations between SBPpeak and several predictors, including sex, age, weight, height, resting systolic blood pressure (SBPrest), resting diastolic blood pressure (DBPrest), resting heart rate (HRrest), peak heart rate (HRpeak), and peak work rate (WR) (all p < .001). The type of sport was also significantly associated with SBPpeak (p < .001), with athletes engaged in mixed and endurance sports showing significant differences in SBPpeak compared to other categories (p < .001).

- **Table S2.** The lower and upper limits of the systolic blood pressure response and the parameters related to workload in male athletes.

| Male |  | ≤9 | 10-11 | 12-14 | 15-17 | 18-30 | 31-40 | 41-50 | 51-60 | 61-70 | >70 |
| --- | --- | --- | --- | --- | --- | --- | --- | --- | --- | --- | --- |
|  | N | 475 | 1,519 | 2,557 | 1,696 | 1,564 | 304 | 339 | 289 | 128 | 26 |
| SBPpeak |  |  |  |  |  |  |  |  |  |  |  |
|  | Mean | 138 | 142 | 155 | 172 | 184 | 188 | 192 | 189 | 186 | 177 |
|  | SD | 16 | 14 | 17 | 20 | 23 | 23 | 24 | 24 | 25 | 21 |
|  | Percentile 05 | 110 | 120 | 130 | 145 | 150 | 150 | 150 | 150 | 140 | 135 |
|  | Percentile 25 | 130 | 135 | 140 | 160 | 165 | 175 | 175 | 170 | 170 | 170 |
|  | Median | 135 | 140 | 150 | 170 | 180 | 190 | 190 | 190 | 190 | 180 |
|  | Percentile 75 | 145 | 150 | 165 | 185 | 200 | 200 | 210 | 210 | 203 | 195 |
|  | Percentile 95 | 160 | 165 | 190 | 210 | 220 | 230 | 230 | 230 | 220 | 205 |
| SBP/W Ratio |  |  |  |  |  |  |  |  |  |  |  |
|  | Mean | 1.1 | 0.9 | 0.8 | 0.6 | 0.6 | 0.6 | 0.7 | 0.8 | 0.9 | 1.1 |
|  | SD | 0.2 | 0.2 | 0.2 | 0.1 | 0.2 | 0.2 | 0.2 | 0.3 | 0.3 | 0.4 |
|  | Percentile 05 | 0.8 | 0.7 | 0.5 | 0.4 | 0.4 | 0.4 | 0.4 | 0.5 | 0.6 | 0.5 |
|  | Percentile 25 | 1.0 | 0.8 | 0.6 | 0.5 | 0.5 | 0.5 | 0.5 | 0.6 | 0.7 | 0.9 |
|  | Median | 1.1 | 0.9 | 0.7 | 0.6 | 0.6 | 0.6 | 0.6 | 0.8 | 0.9 | 1.0 |
|  | Percentile 75 | 1.2 | 1.1 | 0.9 | 0.7 | 0.7 | 0.7 | 0.8 | 1.0 | 1.1 | 1.3 |
|  | Percentile 95 | 1.5 | 1.3 | 1.1 | 0.9 | 0.8 | 0.9 | 1.1 | 1.3 | 1.6 | 2.0 |
| SBP/W Slope |  |  |  |  |  |  |  |  |  |  |  |
|  | Mean | 0.23 | 0.20 | 0.18 | 0.16 | 0.17 | 0.17 | 0.20 | 0.22 | 0.25 | 0.24 |
|  | SD | 0.10 | 0.08 | 0.07 | 0.07 | 0.07 | 0.07 | 0.09 | 0.10 | 0.13 | 0.12 |
|  | Percentile 05 | 0.08 | 0.08 | 0.08 | 0.07 | 0.07 | 0.07 | 0.08 | 0.09 | 0.09 | 0.08 |
|  | Percentile 25 | 0.16 | 0.14 | 0.13 | 0.11 | 0.11 | 0.12 | 0.13 | 0.15 | 0.15 | 0.14 |
|  | Median | 0.22 | 0.19 | 0.17 | 0.15 | 0.16 | 0.17 | 0.18 | 0.21 | 0.22 | 0.22 |
|  | Percentile 75 | 0.30 | 0.25 | 0.22 | 0.20 | 0.21 | 0.22 | 0.24 | 0.27 | 0.31 | 0.30 |
|  | Percentile 95 | 0.40 | 0.34 | 0.31 | 0.29 | 0.30 | 0.30 | 0.36 | 0.40 | 0.52 | 0.40 |
| SBP/(W/kg) Slope |  |  |  |  |  |  |  |  |  |  |  |
|  | Mean | 7.83 | 7.84 | 9.05 | 10.56 | 12.59 | 14.61 | 17.25 | 19.46 | 21.44 | 18.48 |
|  | SD | 3.30 | 3.27 | 3.85 | 4.52 | 5.28 | 6.55 | 8.07 | 9.88 | 12.05 | 10.30 |
|  | Percentile 05 | 2.94 | 3.13 | 3.90 | 4.65 | 5.41 | 5.88 | 6.32 | 6.67 | 5.10 | 4.05 |
|  | Percentile 25 | 5.60 | 5.56 | 6.36 | 7.27 | 8.75 | 10.00 | 11.11 | 12.22 | 11.83 | 10.91 |
|  | Median | 7.45 | 7.50 | 8.57 | 9.62 | 12.00 | 13.60 | 16.00 | 18.04 | 18.92 | 15.56 |
|  | Percentile 75 | 10.00 | 10.00 | 11.11 | 13.16 | 15.56 | 18.27 | 21.67 | 25.00 | 27.05 | 23.67 |
|  | Percentile 95 | 13.33 | 13.51 | 16.22 | 19.15 | 22.22 | 26.67 | 32.43 | 38.00 | 49.50 | 35.29 |

- **Table S3.** The lower and upper limits of the systolic blood pressure response and the parameters related to workload in female athletes.

| Female |  | ≤9 | 10-11 | 12-14 | 15-17 | 18-30 | 31-40 | 41-50 | 51-60 | 61-70 | >70 |
| --- | --- | --- | --- | --- | --- | --- | --- | --- | --- | --- | --- |
|  | N | 113 | 369 | 782 | 521 | 751 | 108 | 155 | 258 | 91 | 27 |
| SBPpeak |  |  |  |  |  |  |  |  |  |  |  |
|  | Mean | 136 | 142 | 153 | 161 | 166 | 168 | 174 | 178 | 178 | 164 |
|  | SD | 14 | 15 | 17 | 16 | 18 | 18 | 20 | 23 | 23 | 23 |
|  | Percentile 05 | 115 | 120 | 130 | 140 | 140 | 140 | 145 | 140 | 130 | 130 |
|  | Percentile 25 | 130 | 130 | 140 | 150 | 150 | 155 | 160 | 160 | 165 | 150 |
|  | Median | 135 | 140 | 150 | 160 | 165 | 170 | 170 | 180 | 178 | 155 |
|  | Percentile 75 | 140 | 150 | 160 | 170 | 180 | 180 | 185 | 190 | 190 | 180 |
|  | Percentile 95 | 165 | 170 | 180 | 190 | 195 | 200 | 215 | 220 | 215 | 205 |
| SBP/WR Ratio |  |  |  |  |  |  |  |  |  |  |  |
|  | Mean | 1.2 | 1.1 | 0.9 | 0.8 | 0.8 | 0.9 | 1.0 | 1.2 | 1.5 | 1.6 |
|  | SD | 0.2 | 0.2 | 0.2 | 0.1 | 0.1 | 0.3 | 0.3 | 0.3 | 0.4 | 0.4 |
|  | Percentile 05 | 0.8 | 0.7 | 0.6 | 0.6 | 0.6 | 0.6 | 0.6 | 0.8 | 0.9 | 0.8 |
|  | Percentile 25 | 1.1 | 0.9 | 0.8 | 0.7 | 0.7 | 0.7 | 0.8 | 1.0 | 1.3 | 1.3 |
|  | Median | 1.2 | 1.1 | 0.9 | 0.8 | 0.8 | 0.9 | 1.0 | 1.2 | 1.4 | 1.6 |
|  | Percentile 75 | 1.4 | 1.2 | 1.0 | 0.9 | 0.8 | 1.0 | 1.2 | 1.4 | 1.7 | 1.8 |
|  | Percentile 95 | 1.7 | 1.5 | 1.2 | 1.1 | 1.0 | 1.6 | 1.6 | 1.8 | 2.2 | 2.2 |
| SBP/WR Slope |  |  |  |  |  |  |  |  |  |  |  |
|  | Mean | 0.24 | 0.23 | 0.20 | 0.19 | 0.19 | 0.22 | 0.28 | 0.32 | 0.35 | 0.29 |
|  | SD | 0.09 | 0.10 | 0.08 | 0.07 | 0.07 | 0.09 | 0.13 | 0.15 | 0.16 | 0.16 |
|  | Percentile 05 | 0.09 | 0.09 | 0.08 | 0.09 | 0.09 | 0.08 | 0.10 | 0.12 | 0.11 | 0.06 |
|  | Percentile 25 | 0.18 | 0.15 | 0.14 | 0.15 | 0.14 | 0.15 | 0.19 | 0.21 | 0.23 | 0.15 |
|  | Median | 0.25 | 0.23 | 0.19 | 0.19 | 0.19 | 0.22 | 0.26 | 0.31 | 0.33 | 0.25 |
|  | Percentile 75 | 0.29 | 0.28 | 0.25 | 0.24 | 0.24 | 0.28 | 0.36 | 0.42 | 0.45 | 0.44 |
|  | Percentile 95 | 0.40 | 0.39 | 0.35 | 0.32 | 0.31 | 0.37 | 0.55 | 0.59 | 0.67 | 0.56 |
| SBP/(WR/kg) Slope |  |  |  |  |  |  |  |  |  |  |  |
|  | Mean | 8.38 | 8.93 | 10.11 | 11.44 | 11.94 | 15.01 | 20.21 | 23.16 | 25.33 | 20.26 |
|  | SD | 3.39 | 3.89 | 4.37 | 4.36 | 4.52 | 6.87 | 10.91 | 11.06 | 12.04 | 13.12 |
|  | Percentile 05 | 3.45 | 3.33 | 4.00 | 5.00 | 5.41 | 5.00 | 5.95 | 7.50 | 8.33 | 3.76 |
|  | Percentile 25 | 6.25 | 6.25 | 7.14 | 8.57 | 8.57 | 9.23 | 12.50 | 14.29 | 16.67 | 8.57 |
|  | Median | 8.57 | 8.57 | 9.46 | 11.11 | 11.43 | 14.00 | 18.18 | 21.82 | 23.53 | 14.71 |
|  | Percentile 75 | 10.00 | 10.94 | 12.50 | 14.29 | 14.86 | 19.31 | 25.58 | 32.07 | 34.68 | 33.33 |
|  | Percentile 95 | 15.00 | 16.00 | 18.75 | 20.00 | 20.00 | 26.92 | 41.18 | 43.75 | 47.06 | 43.21 |

Note: This tables (S2+S3) displays the distribution of peak exercise systolic blood pressure (SBPpeak), SBP to work rate ratios (SBP/WR), and SBP to work rate ratios per kilogram (SBP/(W/kg)) across various age categories for a cohort of athletes. The counts reflect the number of observations within each sex ( Male in Table A2 and Female in Table A3) and age category. Data are summarized by means, standard deviations (SD), and percentile values (5th, 25th, 50th (median), 75th, and 95th percentiles) to provide a detailed view of the distribution characteristics. The age categories are defined in years. These data provide insights into how cardiovascular response to exercise, as indicated by SBP changes relative to power output, varies with age and sex, underpinning the need for age and sex-specific considerations in athletic training and performance evaluation.
